# Forearm elevation impairs local static handgrip endurance likely through reduction in vascular conductance and perfusion pressure: revisiting Rohmert’s curve

**DOI:** 10.1038/s41598-024-83939-7

**Published:** 2025-01-08

**Authors:** L. Heinzl, S. Risse, H. Schwarzbach, O. Hildebrandt, U. Koehler, A. M. Koenig, A. H. Mahnken, R. Kinscherf, W. Hildebrandt

**Affiliations:** 1 Institute for Anatomy und Cell Biology, Department of Medical Cell Biology, Philipps-Universität Marburg, Robert-Koch-Str. 8, 35032 Marburg, Germany; 2 Department of Sleep Medicine, Division of Pneumology, Internal Medicine, University Hospital of Marburg, Philipps-Universität Marburg, Baldingerstr. 43, 35043 Marburg, Germany; 3 Department of Diagnostic and Interventional Radiology, University Hospital of Marburg, Philipps-University, Marburg, Germany

**Keywords:** Skeletal muscle, Static exercise, Fatigue, Blood flow, Gravitation, Hydrostatic pressure

## Abstract

Maximal isometric contraction time (MICT) is critical for most motor tasks and depends on skeletal muscle blood flow at < 40% of maximal voluntary strength (MVC). Whether limb work positions associated with reduced perfusion pressure and facilitated vessel compression affect MICT is largely unknown. In 14 healthy young men we therefore assessed bilateral handgrip MICT at 15, 20, 30, 40, and 70% of MVC in horizontal forearm positions of 0.0, + 27.5 or − 27.5 cm relative to heart level. Forearm blood flow (FBF, venous occlusion plethysmography) and brachial blood pressure were measured repetitively. MICT at 15% MVC was significantly shorter by 66.3 and 86.2 s with forearm position + 27.5 cm (389.6 ± 23.3 s) as compared to 0.0 cm (455.9 ± 34.1 s) and − 27.5 cm (475.8 ± 35.0 s) while MICT at 20–70% MVC was unaffected. Peak FBF at 15% MVC was significantly lower in position + 27.5 cm (11.11 ± 0.92 ml/min/100 ml) compared to 0.0 cm (15.55 ± 0.91 ml/min/100 ml) or − 27.5 cm (14.21 ± 0.59 ml/min/100 ml) and vascular resistance significantly higher in position + 27.5 vs 0.0 or − 27.5 cm. Working position above, but not below heart level may limit MICT at 15% MVC possibly through blood flow reduction arising from increased vascular resistance beside reduced perfusion pressure. Local isometric endurance warrants (re)evaluation regarding hydrostatic/gravitational or other hemodynamic limitations.

## Introduction

Isometric skeletal muscle work is an integral and essential component of most, if not all, physical activities involving postural stabilization of the trunk, the limbs or the head as required for locomotion, standing or sitting. Local fatigue of isometrically working muscle limits work capacity and postural stability and is involved in conditions with chronic back pain^1,2^. There is a well-known non-linear decrease of local isometric muscle endurance with increasing ‘work’ load, as characterized by Rohmert’s curve or related models representing the relationship between the maximal isometric contraction time (MICT) tolerated until task failure by exhaustion and the isometric force in terms of percentage of maximal voluntary contraction (MVC)^3–5^. In the range of up to ~ 60% MVC, isometric work is considered to increase muscle blood flow allowing for, at least in part, aerobic energy supply and prolonged contraction of up to several minutes^4,6^. Thereby, as a major determinant of MICT, local exercise blood flow increase is progressively limited by contraction-induced rise in intramuscular tissue pressure to stable plateaus^7^ that are linearly related to %MVC^8,9^ and thus progressively reduce transmural micro-vascular pressure^4,10^. Such compression pressure imposed on the vessels is only partly compensated for by exercise-induced reflexogenic increases in systemic arterial inflow pressure, especially in the low-pressure sections of the microvasculature^11^. Notably, only at < 15% MVC does muscle blood flow reach an equilibrium and meets metabolic requirements with stable tissue pO_2,_ whereas at ≥ 15% MVC muscle blood flow progressively increases within the first 5 min of contraction and responds with sharp further rise to exercise cessation and related intramuscular pressure release^4,7,10^. In a rather wide, muscle (fiber-)type-dependent range of around 20–70% MVC, transmural pressure is considered to equal and ultimately exceed (increased) intravascular pressure leading to intramuscular vessel collapse and local microcirculatory inhibition or arrest such that remaining blood flow or its experimental occlusion plays no role for MICT^4,6,8,12–14^. Beside O_2_-supply, also elution of potassium, CO_2,_ lactate and other metabolites are prohibited by such ischemic conditions^4,13,15,16^.

Though sports physiology text books commonly present Rohmert’s curve as generally valid^17^, considerable variations of MICT at low levels of %MVC between muscles and conditions have been reported, which could, at least in part, be attributed to factors like gender- or age-related muscle mass/MVC^18,19^, muscle or joint type/location, fiber composition, neuromuscular activation/recruitment or muscle fiber length^5^ as well as the motor task itself^20^. As a main factor, the absolute MVC/muscle mass may to negatively impact MICT via higher intramuscular pressure, explaining, at least in part, lower fatigue resistance in men compared to women and in young compared to older subjects^18,19,21–23^. Beside a ‘pressure-force-relationship’, vessel compression also follows a ‘pressure-depth-relationship’ which, according to Laplace’s law, implies impaired perfusion deep within short bulging as compared to long slender muscles^8,9,24^. Clearly, *experimental* partial or complete occlusion arterial inflow to the working limb muscle effectively reduces MICT at MVC of ≥ 20%^4,6,25,26^ thereby abrogating posture- and gender-related differences^23,27^.

We therefore presently hypothesized, that also *physiological* blood flow limitations, occurring when altering forearm work position, may reduce MICT and ‘flatten’ Rohmert’s curve at low-to-moderate level of %MVC. It has been shown under resting conditions, that passive elevation of the forearm well above atrial level decreases forearm blood flow (FBF) despite a pronounced arteriolar autoregulative (vasodilatative) response to hydrostatic reduction of arterial inflow pressure, because the simultaneous decreases in venous outflow pressure (to around zero) is smaller and the local venous collapse leads to an increase in vascular (postcapillary) resistance^28–33^. In such elevated position, isometric forearm contraction may thus more easily compress (postcapillary) vessels and limit exercise blood flow in comparison to lower work positions. However, surprisingly, studies addressing such gravitational/hydrostatic effect on local endurance are rare and do not cover Rohmert’s curve. A single small early study (n = 4) reported a reduction in both, exercise blood flow and MICT at 25% and 40% MVC of the forearm placed above heart (while supine) compared to heart level (while sitting)^27^. This effect was abolished under ischemic conditions, however, the role of trunk position vs. relative forearm position in the local blood flow limitations could not be separated.

We therefore presently ‘re-visited’ the complete curve by Rohmert and assessed the effect of changes in work positions between 0.0 cm (reference position), + 27.5 cm (elevated), and − 27.5 cm (lowered) relative to the 3rd parasternal intercostal space on MICT and FBF in young lean healthy men without vascular risks. We found that MICT was significantly by > 1 min shorter and FBF massively reduced at 15% MVC in the position + 27.5 cm as compared to the positions 0.0 and − 27.5 cm.

## Methods

### Study population and design

Fourteen young healthy male volunteers were recruited by public announcement to the present study which was approved by the Ethical Committee of the University of Marburg (35/17; May 2nd, 2017) and performed according to the amended Declaration of Helsinki. All participants gave their oral and written informed consent to participate in the present study.

Exclusion criteria were: Age < 18 or > 35, present or former smoking habit, blood donation or exposure to an altitude > 2000 m within the last six months, enrolment in competitive sports programs (> 6 h per week), arterial hypertension (RR systolic > 140 mmHg, diastolic, > 100 mmHg) or hypotension (RR systolic < 90 mmHg), any history/ prior events, symptoms, or known traditional risks of cardiovascular disease, any major intestinal, hepatic, renal, neurological or psychiatric or orthopedic disease, musculoskeletal injury of the upper limb within the last year, any alcohol or drug abuse, any medication or antioxidant supplementation including N-acetylcysteine within the last 3 months, insufficient cooperation in or tolerance with regard to the experimental setting, and missing oral or written consent.

Health assessment before inclusion into the study comprised the subject’s medical history including regular physical activity, a physical examination, routine laboratory venous blood parameters covering traditional vascular risk factors like the lipid status, HbA1c and uric acid, bilateral blood pressure measurement in sitting position, pulmonary function, and a 12-lead electrocardiogram (ECG) at rest. Vascular risk assessment furthermore included measurement of right and left intima-media-thickness (IMT) by the US-System, Logiq E9 with a 7.5 MHz scanner 9D-L (GE, Connecticut, USA, Software Version 3.1.2.).

Sample size of n ≥ 13 was calculated to detect a significant difference (Cohens’ d = 0.85, power = 0.8, p ≤ 0.05) of MICT between forearm positions + 27.5 and 0.0 cm (reference position) or − 27.5 cm relative to the 3rd ICS. All measurements were conducted under fasting conditions of > 4 h abstinence from any oral intake except for water.

### Forearm water displacement volume and MRT volumetry of muscle and fat

The water displacement volume and the maximal circumference of the right and left forearm were obtained between the olecranon and the processus styloideus radii. Moreover, within this section of both forearms, bilateral volume and maximal cross-sectional area (CSA) of all forearm muscles were determined by 1.5 T-magnetic resonance (MR) scanner (Siemens Espree, Erlangen, Germany). Serial cross-sectional images were acquired using a T2 turbo-spin-echo sequence (TSE, TR = 5490 ms, TE = 91 ms, slice thickness = 4.0 mm, interslice gap = 0%). The software OSIRIX (OSIRIX v.6.0.2 32-bit, Pixmeo, Bernex, Switzerland) with segmentation plugin (Segmentation 1.0.xr plugin, Chimaera GmbH, Erlangen, Germany) was used for the volugraphy, which also included T2-weighted bilateral imaging and volugraphy of subcutaneous fat in all volunteers. Forearm muscle CSA at maximal forearm CSA (the site of plethysmography) was determined with subtraction of ulna, radius and vascular embedding CSA for all muscles enclosed as well as for those considered to effectively participate in handgrip movement. Thereby, extensor muscles contributing to this movement were included into this measurement, while muscles not involved in closing the fist like the brachioradialis, pronator teres, and supinator muscle were excluded.

### Relative forearm handgrip work load and positioning

**Fig. 1:**
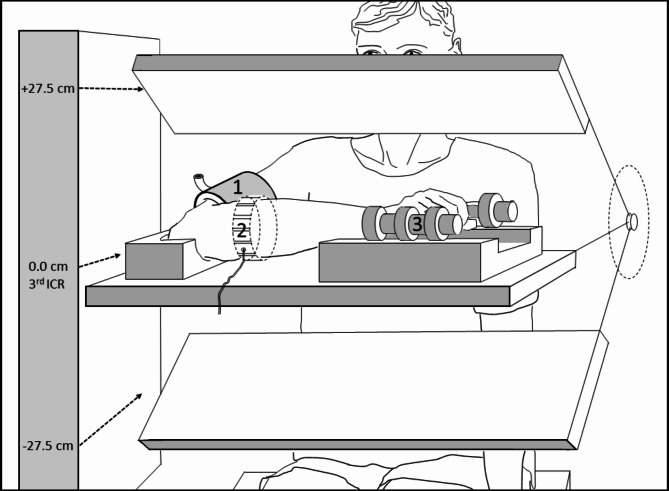
Experimental setup showing the horizontal forearm support of the sitting subject in the three different experimental olecranon positions of 0.0 cm (reference position), + 27.5 cm (elevated), and − 27.5 cm (lowered) relative to the parasternal 3rd intercostal space (ICR). Venous occlusion plethysmography was performed with an upper arm occlusion cuff (1) and a linked-chain mercury-in-silastic-strain-gauge sensor (2) placed around the maximal forearm circumference as indicated for the right arm. Isometric handgrip was performed by a supported dynamometer (3) with visual feedback. Brachial blood pressure was measured at the contralateral dependent non-exercising arm (for details see Methods section).

To study postural (hydrostatic) effects on Rohmert’s curve intra-individually, subjects right and left local handgrip endurance was assessed in terms of MICT tolerated at 15%, 20%, 30% 40% and 70% of individual MVC using a hydraulic hand dynamometer (Baseline™, Fabrication Enterprise, Elmsfrod, NY, USA) with analog output for PC-based feedback and data storage. The three forearm (olecranon) test positions defined as − 27.5 cm, 0.0 cm (reference position), + 27.5 cm relative to the parasternal 3rd ICS were defined by an adjustable pivotable metal platform mounted to an experimental seat (Fig. 1). Subjects resumed a comfortable upright sitting test position with 90° elbow flexion and pronation of the forearm, which was supported in a ventral, transverse, horizontal position (with regard to the ulna) with wrist and elbow pads in all the three test positions. Isometric handgrip contractions were performed using handgrip dynamometer, with the handgrip dynamometer loosely fitted into a special foam pad to exclude the impact of mechanical forces other than through handgrip exertion. Subjects were asked and initially trained to control the required exact experimental %MVC by a feed-back track presented on a monitor with indication of a ± 2.5% range. The contraction force developed at each %MVC level was electronically tracked, stored and analyzed for the exact MICT tolerated within the required range of %MVC. Maximal tolerable MICT at each %MVC level was assessed as task failure, defined as handgrip force > 2.5% below the target force for > 1 s, which in all cases coincided with the inability to continue contraction despite strong verbal encouragement and a BORG rating of ≥ 19.

### Forearm vascular function

All vascular forearm measures were obtained by venous occlusion plethysmography (VOP) using the Compactus 732 system (Sogut, Geretsried, Germany) with its self-calibrating, linked-chain, mercury-in-silastic-strain-gauge sensor placed around the maximal forearm circumference and automated rapid inflation (0.5 s) of an upper arm occlusion cuff.

### a) Forearm blood flow (FBF) and vascular resistance

**Fig. 2:**
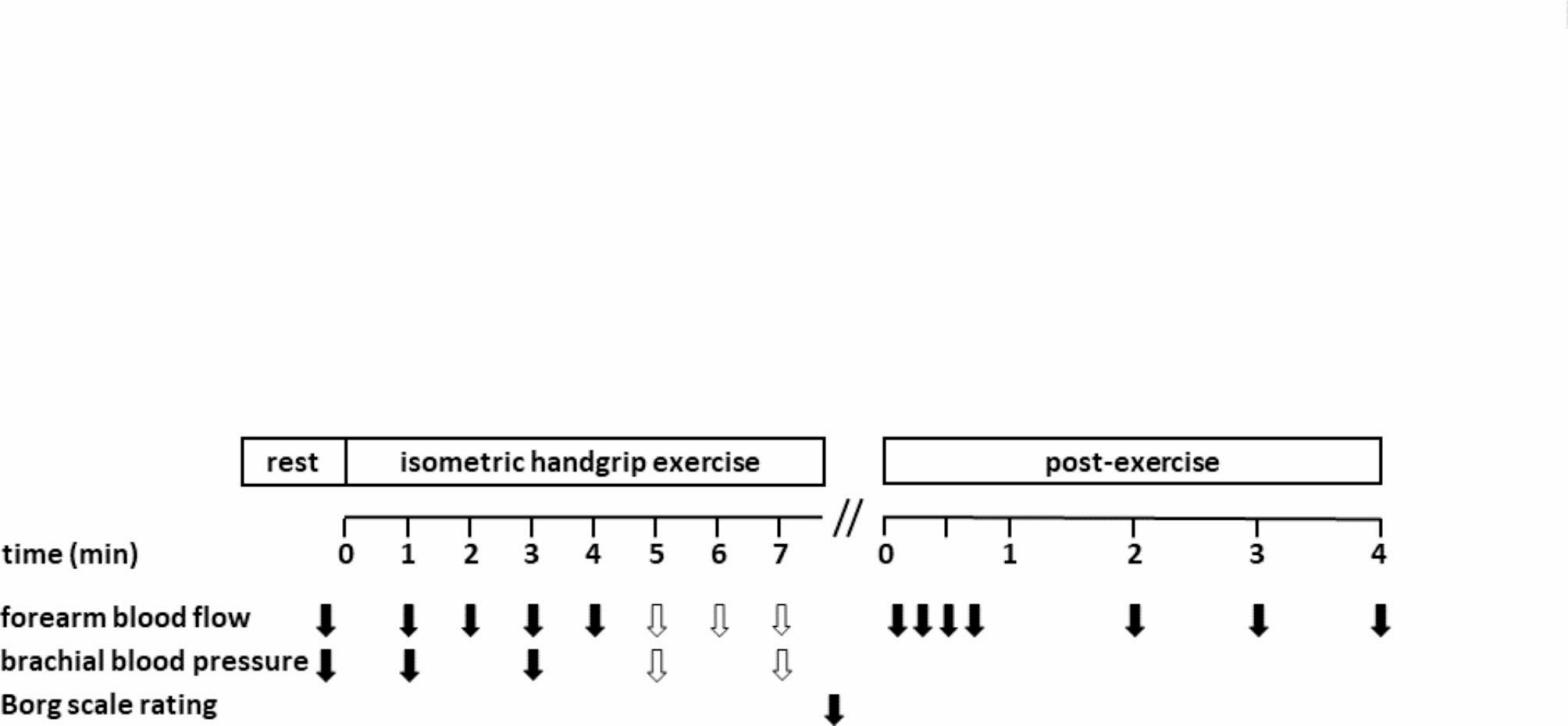
Principal time schedule of pre-, intra- and post-exercise measurements of forearm blood flow and brachial blood pressure as well as Borg scale self-rating. For example, the isometric workload of 15% MVC could be sustained for 4 min by all subjects (as indicated by filled arrows). Statistical analysis of forearm blood flow and blood pressure values considered for this interval only, since longer maximal isometric contraction times were tolerated only by subgroups (as indicated by open arrow).

Segmental FBF (ml/min/100 ml tissue) was calculated by the VOP device from the initial (6 s) forearm volume changes (pulsatile blood inflow) prompted by venous occlusion via rapid proximal cuff inflation to 60 mmHg. Since room air was kept at 21 °C no arterial occlusion cuff at the wrist (causing discomfort during handgrip exercise) was used to exclude hand circulation according to^34,35^. VOP has been validated for FBF measurement during isometric muscle contraction^4,35,36^. Resting blood flow was assessed in triplicate every 54 s. The time protocol for repetitive single FBF measurements during and after isometric contractions at various % MVC is presented in Fig. 2. Post-occlusion peak blood flow (arterial reserve) at baseline was determined as the maximum of 5 recordings every 10 s immediately following 5 min of arterial occlusion (> 200 mmHg) in forearm position 0.0 cm (reference position) relative to the 3rd ICS only.

The local forearm vascular resistance at rest and after 2 min of isometric contraction was calculated as the ratio of mean forearm perfusion pressure (the difference between local mean arterial inflow pressure and estimated venous outflow pressure) to simultaneous FBF readings. Local mean arterial inflow pressure was calculated as the local mean arterial pressure (MAP = diastolic pressure + 1/3 (systolic pressure − diastolic pressure)) assessed by the validated automated oscillometric device Omron HEM 705 CP (Omron healthcare, Kyoto, Japan) at the dependent non-working contralateral upper arm with corrections for the hydrostatic pressure changes caused by forearm elevation to + 27.5 cm (− 20.23 mmHg) or forearm lowering to − 27.5 cm (+ 20.23 mmHg) from 0.0 cm (reference position) relative to the 3rd parasternal ICS, respectively. Thereby, venous outflow pressure was assumed to be unchanged (around 0.0 mmHg) with the position + 27.5 cm, while lowering the forearm to − 27.5 cm was considered to increase hydrostatic local arterial inflow or venous outflow pressure equally by 20.23 mmHg. As, realistically, venous outflow pressure in the forearm position of 0.0 cm (reference position) relative to the parasternal 3rd ICS could be assumed to vary between 0 and 10 mmHg (and to slightly exceed central venous pressure (CVP)), vascular resistance was calculated assuming a venous outflow pressure equal to either 0.0 mmHg or, alternatively, 10 mmHg, with this range approximately covering prevailing anatomical-physiological variations.

### b) Forearm venous compliance

Right and left side measures were taken in forearm position 0.0 cm (reference position) relative to the 3rd ICS according to the automated VOP device program in terms of forearm volume changes within 5 min (complete vascular filling) following an upper arm cuff occlusion pressure application of 80 mmHg.

### c) Forearm capillary filtration rates (CFR) and capillary filtration capacity (CFC)

Bilateral CFRs covering 5 min after complete vascular filling (min 6–9) were determined separately for four different upper arm cuff venous occlusion pressures which were 30, 40, 60, and 80 mmHg in forearm position 0.0 cm (reference position) relative to the 3rd ICS. From these data, CFCs were individually calculated as the slope (ml min^−1^ 100 ml^−1^ mmHg^−1^) of the linear regression line across the CFRs obtained with these four different occlusion pressures; CFC yields an indirect measure capillary exchange capacity (surface * permeability)^37,38^.

### Randomization of test order

Individual MICTs were assessed in duplicate (for the right and left forearm) for each of the five work loads of 15%, 20%, 30%, 40% and 70% of MVC (100%) all of which were compared between the three forearm working positions defined as − 27.5 cm, 0.0 cm (reference position) and + 27.5 cm relative to the 3rd ICS. In order to control for and minimize the confounding effects of training or fatigue arising from repetitive testing, the order of these overall 15 unilateral (30 bilateral) single isometric contraction tests were randomized with regard to the order of both, the 5 levels of %MVC and the 3 forearm positions as follows: From the permutations according to the cartesian product of % MVC levels and forearm position, only those test series were randomly chosen that rendered maximal cumulative difference between subsequent forearm position or handgrip %MVCs. The resulting individual order of 15 test condition was used for bilateral testing with the left/right order being individually fixed but alternating between subjects. Any consecutive tests were separated by at least 15 min, any unilateral consecutive tests were separated by at least 30 min. The overall 30 tests were performed at the same time of the day on four separated test days (3–4 unilateral/6–8 bilateral tests per test day) within 2 weeks.

### Statistics

Data were presented as mean ± standard error of the mean (SEM) covering the total study population for all baseline parameters as well as for each experimental condition of (five different %MVC levels in three forearm positions) and time point of measurement. Significant intra-individual differences between forearm positions or between time points were detected by the paired Student’s t test or, if values were not normally distributed according to the Kolmogorow–Smirnow-test, by the non-parametric Wilcoxon test for paired observation. Bivariate linear correlations between individual measures were presented by Pearson’s correlation coefficient r, and the p values. A p < 0.05 was considered as statistically significant. Given the explorative, hypothesis-generating nature of the study, no Bonferroni correction was intended. All statistical procedures were performed by SPSS (version 27, IBM Munich, Germany). All graphs were created by means of GraphPad Prism-8 software (GraphPad Software Inc. San Diego, California, US).

## Results

All young, male, non-obese study participants (Table 1) were right-handed and accordingly had a slightly but significantly smaller left than right maximal forearm circumference, a by 6% smaller left than right CSA with regard to both, muscles in total as well as the subset of handgrip-involved muscles enclosed in MRT-related maximal CSA of the forearm. This translated into a by 6–9% lower left than right handgrip MVC in all test positions. A significant correlation existed between the handgrip-involved muscle CSA and MVC at baseline for both, the right (r = 0.653, p = 0.011) and left (r = 0.589, p = 0.027) forearm.

**Table 1:** Epidemiological data, bilateral forearm volume and muscle cross-sectional area as well as handgrip strength while standing and in the three test positions.

			Mean ± SE	Range
Age	years		23.5 ± 0.7	21–31
Body height	cm		183.3 ± 1.7	172–192
Body weight	kg		80.1 ± 2.4	69–99
BMI	kg m^−2^		23.8 ± 0.5	20.8–28.2
Forearm circumference (maximum)	cm	Right	28.4 ± 0.4	26.0–30.4
		Left	27.7 ± 0.4 $$$	25.0–29.5
Forearm volume (H_2_0 displacement)	cm^3^	Right	1204 ± 38	1020–1430
		Left	1172 ± 41	867–1400
CSA_max_ of all forearm muscles (1.5 T-MRT)	mm^2^	Right	4054 ± 130	3379–4782
		Left	3830 ± 116 $$$	3369–4667
CSA_max_ of all handgrip muscles (1.5 T-MRT)	mm^2^	Right	2703 ± 93	2157–3161
		Left	2536 ± 106 $	2014–3194
Handgrip MVC (baseline, standing)	N	Right	57.0 ± 2.6	44–77
		Left	52.1 ± 2.3 $$	42–68
Handgrip MVC (test position + 27.5 cm)	N	Right	56.2 ± 2.4	44–72
		Left	53.4 ± 2.1 $	45–70
Handgrip MVC (test position 0.0 cm)	N	Right	57.1 ± 2.4	45–74
		Left	53.4 ± 2.1 $$	46–71
Handgrip MVC (test position −27.5 cm)	N	Right	54.6 ± 2.5	40–69
		Left	51.4 ± 1.8 $	43–65

BMI = body mass index; CSA_max_ = maximal cross-sectional forearm area; ‘all forearm muscles’ = all forearm muscles enclosed at CSA_max_; ‘all handgrip muscles’ = ‘all forearm muscles’ at CSA_max_ with exclusion of the brachioradialis, pronator teres and supinator muscles; MVC = maximal voluntary contraction; ^$^ p < 0.05, ^$$^ p < 0.01, ^$$$^ p < 0.001 left vs. right side by paired t-test or Wilcoxon test.

Moreover, there were highly significant, close correlations between the left and right side regarding the muscle CSA in total (r = 0.945, p < 0.001), the handgrip-relevant muscle CSA (r = 0.934, p < 0.001) and the MVC while standing at baseline (r = 0.889, p < 0.001) or when sitting with forearm at 0.0 cm reference position (r = 0.879, p < 0.001). Handgrip MVC values ranged well within the reference interval reported for men of this body height and age group^39^. Notably, left or right handgrip MVC did significantly differ neither between the standing position (at baseline, with dependant forearm) and the three forearm test positions + 27.5 cm, 0.0 cm (reference position) or − 27.5 cm while seated, nor between any of these three experimental forearm positions.

Regarding their vascular risk profile (Table 2), all volunteers had normal bilateral brachial blood pressure and postabsorptive venous plasma lipids, HbA1c, Uric acid, TNF and C-reactive-protein. This was also true for their hemoglobin concentration and hematocrit as well as for all other routine hemogram parameters (not shown).

**Table 2:** Vascular vascular profile and hemogram parameters of the young male study population.

			Mean ± SE	Range
BP systolic	mmHg	Right	118.3 ± 3.3	95–138
		Left	117.6 ± 3.2	100–139
BP diastolic		Right	76.9 ± 2.8	60–97
		Left	74.7 ± 2.7 $	61–96
IMT	mm	Right	0.47 ± 0.03	0.30–0.63
		Left	0.44 ± 0.02 $	0.30–0.65
Triglycerides	mg 100 ml^−1^		100.0 ± 13.4	37–205
Total cholesterol	mg 100 ml^−1^		177.0 ± 8.2	127–247
LDL	mg 100 ml^−1^		109.0 ± 6.6	68–170
HDL	mg 100 ml^−1^		57.5 ± 2.8	42–78
HbA1c	%		5.1 ± 0.1	4.3–5.4
Uric acid	mg 100 ml^−1^		5.7 ± 0.3	4.2–7.4
TNF-α	pg ml^−1^		5.4 ± 0.3	4.1–8.4
C-reactive protein	mg l^−1^		0.82 ± 0.2	0.20–2.30
Hemoglobin	g 100 ml^−1^		15.2 ± 0.2	14.2–16.4
Hematocrit	%		43.9 ± 0.5	41–47

BP = arterial blood pressure, IMT = intima-media-thickness; LDL = low density lipoprotein; HDL = high density lipoprotein. Hb A1C = glycated hemoglobin (glycohemoglobin); TNF-α = tumor necrosis factor-α. $ p < 0.05 left vs. right side by paired t-test or Wilcoxon test.

Vascular function parameters (Table 3) were well comparable between the right and left forearm regarding resting blood flow, post-occlusion peak FBF, venous compliance, (trans-) capillary filtration rates at 30, 40, 60 or 80 mmHg occlusion pressures and calculated filtration capacity as an indirect measure capillary exchange capacity (surface * permeability)^38^.

**Table 3:** Vascular function parameters of left and right forearm at baseline in test position 0.0 cm relative to 3rd parasternal intercostal space (ICR).

			Mean ± SE	Range
Resting FBF	ml min^−1^ 100 ml^−1^	Right	2.93 ± 0.38	0.94–6.24
		Left	3.18 ± 0.34	1.24–5.74
Post-occlusion peak FBF	ml min^−1^ 100 ml^−1^	Right	24.78 ± 1.16	16.20–32.50
		Left	26.51 ± 1.70	14.90–40.80
Venous compliance (80 mmHg)	ml 100 ml^−1^ mmHg^−1^	Right	3.65 ± 0.29	1.80–5.30
		Left	3.88 ± 0.22	2.30–5.00
Capillary filtration rate at 30 mmHg	ml min^−1^ 100 ml^−1^	Right	0.0229 ± 0.0107	0.0000–0.1200
		Left	0.0229 ± 0.0098	0.0000–0.1000
At 40 mmHg	ml min^−1^ 100 ml^−1^	Right	0.0629 ± 0.0105	0.0000–0.1200
		Left	0.0543 ± 0.0100	0.0000–0.1200
At 60 mmHg	ml min^−1^ 100 ml^−1^	Right	0.1586 ± 0.01972	0.0600–0.3600
		Left	0.1531 ± 0.0235	0.0300–0.3800
At 80 mmHg	ml min^−1^ 100 ml^−1^	Right	0.1785 ± 0.0182	0.1000–0.0360
		Left	0.1554 ± 0.0182	0.0600–0.3400
Capillary filtration capacity (CFC)	ml min^−1^ 100 ml^−1^ mmHg^−1^	Right	0.00327 ± 0.00035	0.0005–0.0052
		Left	0.00317 ± 0.00032	0.0010–0.0051

FBF = forearm blood flow; Resting FBF data represent mean of 3 measures every 60 s taken after 30 min sitting rest. Post-occlusion peak FBF was assessed as maximum of 5 recordings every 10 s after 5 min suprasystolic arterial occlusion. Venous compliance was determined as vascular volume increase within 5 min after 80 mmHg venous occlusion pressure. Capillary filtration rates were measured as transcapillary filtrative volume changes recorded covering min 6–9 after application of the indicated venous occlusion pressure. Capillary filtration capacity was individually calculated as the slope of increase in filtration rates per mmHg venous occlusion pressure between 30–80 mmHg. All measures were obtained at baseline by venous occlusion plethysmography at maximal forearm circumference. No significant differences were detected between right and left forearm.

As shown in Table 4, changes in right or left MVC across each of the four test days (which covered 3–4 out of total 15 randomized bilateral test conditions each) ranged between 0.4 and −5.8% and reached significance only in case of left MVC on test day 1. Changes in right or left MVC between any of the four test days ranged between 0.1% and 2.7% were all non-significant (Table 4).

**Table 4:** Maximal handgrip strength (MVC) before and after each of four test-days (with subjects standing with dependent arm).

		Test day		Pre-test	Post-test
MVC	N	1	Right	56.21 ± 2.57	54.85 ± 1.95
			Left	52.53 ± 2.44	50.38 ± 2.01 §
		2	Right	57.00 ± 2.32	55.14 ± 1.50
			Left	51.71 ± 2.34	51.57 ± 1.56
		3	Right	55.50 ± 2.12	54.21 ± 1.6
			Left	50.71 ± 1.73	50.92 ± 1.53
		4	Right	57.75 ± 2.38	54.41 ± 1.94
			Left	52.58 ± 2.03	50.83 ± 1.52

Data are mean ± SEM. § p < 0.05, post- vs- pre-test by paired *t*-test/ Wilcoxon test.

MICT at 15%, 20%, 30%, 40%, and 70% MVC were comparable and not significantly different between right and left forearm in all test positions + 27.5, 0.0 (reference position) and − 27.5 cm relative to the 3rd ICS (not shown). In view of the small (6%) difference between left and right handgrip-relevant muscle mass (Table 1) and well comparable left and right vascular function (Table 3), left and right MICTs were considered as duplicate measures and averaged to represent Rohmert’s curve between 15 and 100% MVC for comparison between the three test positions + 27.5 cm, 0.0 cm (reference position) and − 27.5 cm.

**Fig. 3:**
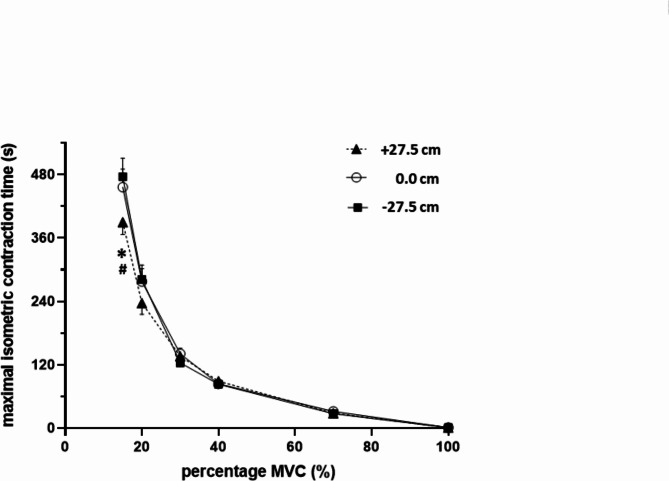
Relationship between maximal isometric contraction time tolerated until task failure (MICT) and graded % MVC (Rohmert’s curve) presented separately for the forearm test positions + 27.5, 0.0, and − 27.5 cm relative to the parasternal 3rd intercostal space (as indicated within the graph). * for p < 0.05 + 27.5 cm vs 0.0 cm; # for p < 0.05 + 27.5 cm vs − 27.5 cm.

As a main finding (Fig. 3), MICT at 15% MVC was found to be significantly by 66.3 s and by 86.2 s shorter in position + 27.5 than in the positions 0.0 cm (reference position) and − 27.5 cm, respectively; MICT at 20% MVC showed a respective reduction by 40.6 and 44.5 s, however, this difference failed to reach statistical significance. At the higher relative work load levels of 30%, 40%, and 70% MVC, MICT was virtually identical between all three forearm test positions.

**Fig. 4:**
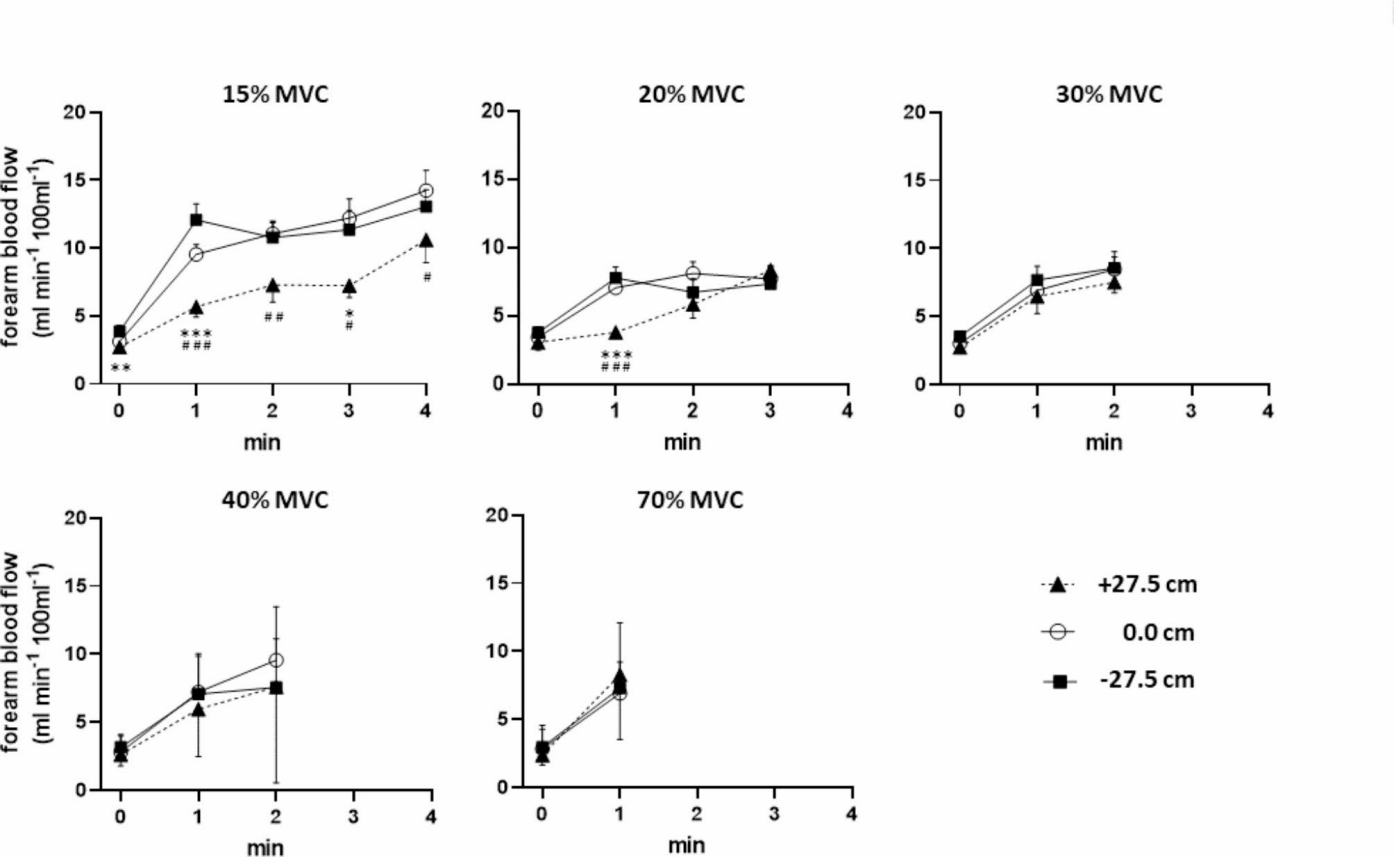
Response of forearm blood flow to isometric exercise in the three different positions + 27.5, 0.0, and − 27.5 cm relative to the parasternal 3rd intercostal space with the five levels of % MVC as indicated: 15% (upper let panel), 20% (upper middle panel), 30% (upper right panel), 40% (lower left panel), and 70% (lower right panel). Forearm blood flow values during exercise significantly exceeded those at rest in all cases. * for p < 0.05, ** for p < 0.01, *** for p < 0.001 + 27.5 cm vs 0.0 cm; # for p < 0.05, ## for p < 0.01, ### for p < 0.001 + 27.5 cm vs − 27.5 cm.

Upon static exercise at 15% MVC (Fig. 4, upper left panel), FBF (mean of left and right values) more than doubled within the 1st min in all forearm positions but reached a significantly lower level in forearm position + 27.5 cm as compared to 0.0 cm (reference position) or − 27.5 cm. The difference in FBF between 27.5 cm and 0.0 cm (reference position) persisted until the 4th min whereby values progressively increased in parallel. FBF in forearm position − 27.5 cm did not significantly differ from that in 0.0 cm (reference position) and was significantly higher compared to position + 27.5 cm in the 3rd min. Upon static exercise at 20% MVC (Fig. 4, upper middle panel), the initial FBF response was massively reduced compared to 15% MVC, however, reached significantly lower level in position + 27.5 than 0.0 cm (reference position) and − 27.5 cm. Thereafter, FBF were similar between all three positions within the shorter (3 min) observation interval, showing a linear FBF increase for position + 27.5 only. Within the 2 min or 1 min observation interval at 30% MVC (Fig. 4, upper right panel), 40% MVC (Fig. 4, lower left panel) or 70% MVC (Fig. 4, lower right panel), respectively, no position-related differences in FBF were observed. Moreover, no further MVC level-related reduction in the initial steep (1st min) or in the slow consecutive increase (1st–2nd min) was found as compared to 20% MVC. Notably, no complete inhibition of FBF or reduction below resting value was observed with high % MVC levels of 30, 40, or 70% or with any forearm position.

Accordingly, individual peak FBF (Table 5) reached during static exercise at 15% MVC were significantly by 29% and 22% reduced in forearm position + 27.5 cm as compared to 0.0 cm (reference position) and − 27.5 cm, respectively. Likewise, with static exercise at 20% MVC the individual peak FBF were found to be significantly by 27% and 25% lower in forearm position + 27.5 cm as compared to 0.0 cm (reference position) and − 27.5 cm, respectively. This significant peak FBF reduction through forearm elevation disappeared with further work load increase (and lower peak FBF) with higher MVC, except for a 26% reduction at + 27.5 cm vs. 0.0 cm (reference position) at 40% MVC. Overall, increasing static work load from 15% MVC to 30%, 40% or 70% MVC significantly and progressively reduced peak FBF in forearm position 0.0 cm (reference position) and − 27.5 cm, while in forearm position + 27.5 cm this was the case only at 40% MVC.

**Table 5:** Forearm blood flow (FBF, mean of left and right side) in the three working positions: resting values and individual maxima reached during and immediately after isometric contraction.

Forearm working position relative to 3rd ICS			− 27.5 cm	0.0 cm	+ 27.5 cm
Resting FBF pre-exercise	ml min^−1^ 100 ml^−1^	All %MVC	3.45 ± 0.28	3.06 ± 0.18	2.69 ± 0.12*##
Peak FBF intra-exercise	ml min^−1^ 100 ml^−1^	15% MVC	14.21 ± 0.59	15.55 ± 0.91	11.11 ± 0.92***##
		20% MVC	13.30 ± 0.97	13.67 ± 0.86	10.01 ± 0.86**#
		30% MVC	10.95 ± 0.84 !!!	11.16 ± 0.48 !!!	10.76 ± 0.87
		40% MVC	9.20 ± 0.81* !!!	11.11 ± 0.92 !!!	8.17 ± 1.26** !
		70% MVC	8.38 ± 2.92 !!	7.18 ± 0.78 !!!	8.76 ± 0.99
Peak FBF post-exercise	ml min^−1^ 100 ml^−1^	15% MVC	13.78 ± 1.02**	16.15 ± 1.20	13.83 ± 1.39**
		20% MVC	13.29 ± 0.88***	18.44 ± 1.35	13.93 ± 0.81**
		30% MVC	13.27 ± 0.86***	17.31 ± 1.14	15.90 ± 0.99 ##
		40% MVC	13.25 ± 0.72***	19.70 ± 2.55	14.77 ± 1,07
		70% MVC	14.24 ± 0.82***	17.23 ± 0.71	15.37 ± 0.72

Data are mean ± SEM. FBF = forearm blood flow;

* p < 0.05, ** p < 0.01, *** p < 0.001 vs 0.0 cm forearm working position,

# p < 0.05, ## p < 0.01, ### p < 0.001 vs. −27.5 cm forearm working position,

! p < 0.05, !! p < 0.01, !!! p < 0.001 vs 15% MVC by paired t-test/ Wilcoxon.

Systolic and diastolic brachial blood pressure (Table 6) at rest and after 2 min of exercise at 15% and 20% MVC did not significantly differ between the three forearm positions, however, significantly increased with exercise at 15% and 20% MVC in all positions, thereby reaching the highest systolic values in forearm position + 27.5 cm.

**Table 6:** Brachial blood pressure at rest and after 2 min exercise in the three working positions.

				− 27.5 cm	0.0 cm	+ 27.5 cm
Brachial blood pressure (mmHg)	15% MVC	Rest	Systolic	121.6 ± 2.8	122.0 ± 3.3	117.2 ± 5.1
			Diastolic	75.3 ± 3.0	76.5 ± 1.5	78.2 ± 1.8
		2 min exercise	Systolic	134.3 ± 4.5 ^§§§^	134.5 ± 3.8 ^§§§^	136.1 ± 4.3 ^§§§^
			Diastolic	90.2 ± 3.3 ^§§§^	90.4 ± 2.9 ^§§§^	89.8 ± 2.7 ^§§§^
	20% MVC	Rest	Systolic	121.1 ± 3.0	122.1 ± 2.8	123.5 ± 2.9
			Diastolic	79.8 ± 2.0	77.1 ± 2.0	79.8 ± 1.7
		2 min exercise	Systolic	133.2 ± 6.1 ^§§^	136.6 ± 6.7 ^§§^	157.3 ± 3.2 ^§§§^
			Diastolic	94.7 ± 5.2 ^§§§^	89.6 ± 4.5 ^§§^	97.6 ± 3.4 ^§§§^

Data are mean ± SEM. *FBF* forearm blood flow.

§ p < 0.05, §§§ p < 0.001 exercise (2nd min) vs. rest.

Note that no blood pressure data were obtained with contraction times < 2 min (MVC > 20%).

The individual post-exercise peak FBF, observed within 2 min after release of contraction-induced intramuscular pressure revealed no difference between the 5 levels of % MVC within each of the forearm position. However, post-exercise peak FBF at 15% and 30% MVC were significantly lower with both, forearm position + 27.5 (− 14% and − 24%, respectively) as well as forearm position − 27.5 cm (− 15% and − 28%, respectively) compared to 0.0 cm (reference position), with this pattern observed for higher % MVC levels in the lower forearm position of − 27.5 cm.

**Fig. 5:**
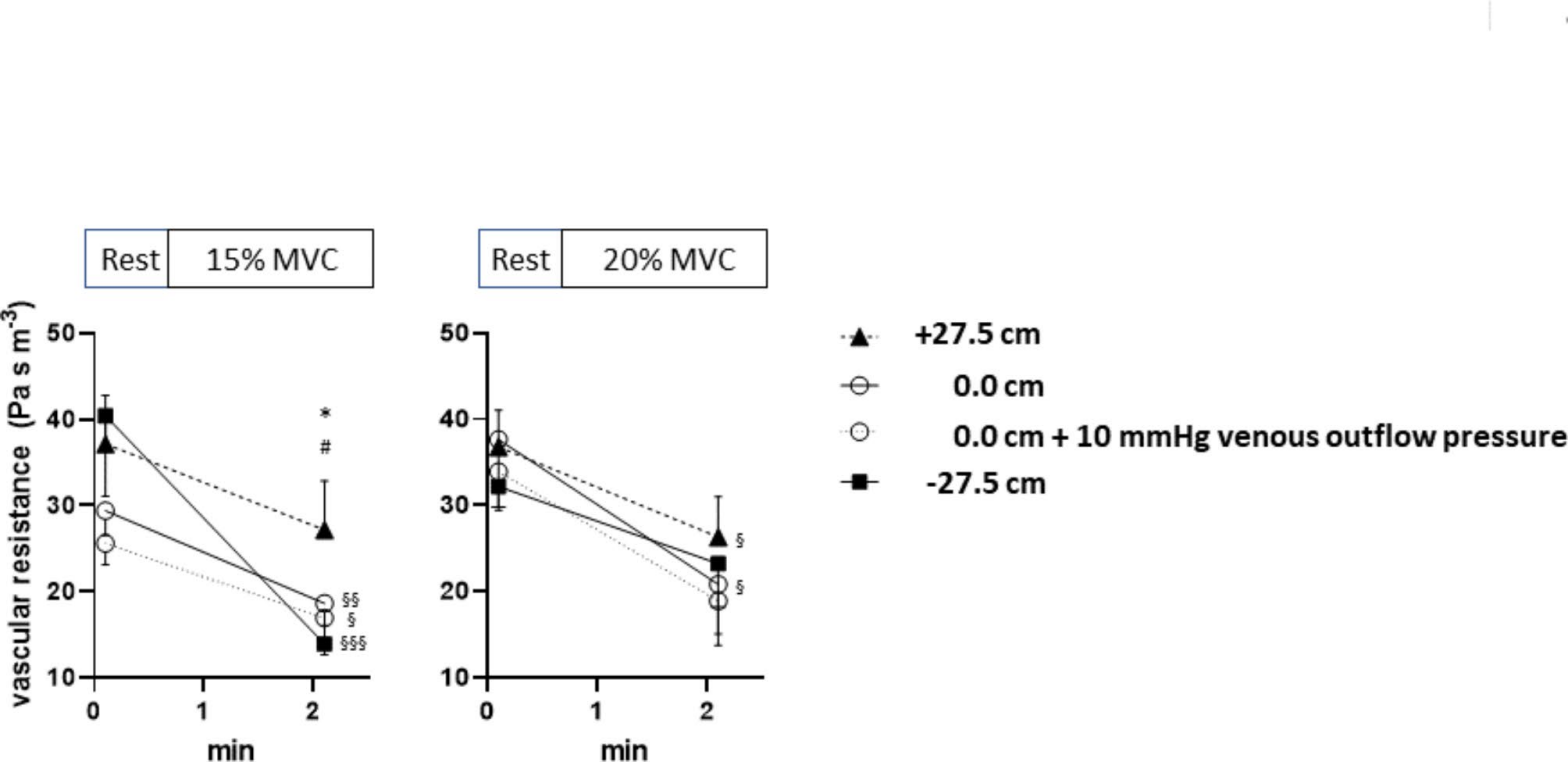
Forearm vascular resistance calculated as the ratio between estimated mean forearm perfusion pressure and simultaneous forearm blood flow readings at rest and after 2 min of isometric contraction at 15% MVC (left panel) and 20% MVC (right panel) in the forearm position + 27.5, 0.0, and − 27.5 cm, as indicated. Mean forearm perfusion pressure was estimated as the difference between local mean arterial inflow pressure and venous outflow pressure. Local mean arterial inflow pressure was calculated as the brachial mean arterial pressure (MAP = diastolic pressure + 1/3 (systolic pressure − diastolic pressure)), derived from oscillometric measures from the dependent non-working contralateral upper arm with corrections for the hydrostatic pressure changes caused by forearm elevation to + 27.5 cm (− 20.23 mmHg) or forearm lowering to − 27.5 cmH_2_O (+ 20.23 mmHg) from 0.0 cm (reference position) relative to the parasternal 3rd ICS, respectively. Thereby, venous outflow pressure was assumed to be unchanged (around 0.0 mmHg) with forearm elevation to + 27.5 cm, while lowering the forearm to − 27.5 cm was considered to increase hydrostatic local arterial inflow or venous outflow pressure equally by 27.5 cmH_2_O (20.23 mmHg). As, realistically, venous outflow pressure in the forearm position of 0.0 cm (reference position) relative to the parasternal 3rd ICS could be assumed to vary between 0 and 10 mmHg (and to slightly exceed central venous pressure), vascular resistance was calculated assuming a venous outflow pressure equal to either 0.0 mmHg or, alternatively, 0.7355 mmHg, with this range approximately covering prevailing anatomical-physiological variations. * for p < 0.05 + 27.5 cm vs 0.0 cm or 0.0 cm + 10 mmHg; # for p < 0.05 + 27.5 cm vs −27.5 cm; § for p < 0.05, §§ for p < 0.01, §§§ for p < 0.001 exercise (2nd min) vs. rest.

Estimations of the local vascular resistance (Fig. 5) were based on FBF before or 2 min after exercise start and on simultaneous brachial blood pressure readings (for calculation of local perfusion pressure as the difference between mean arterial pressure and estimated venous pressure) from the non-working arm, where cuff position represented about the hydrostatic level of the 0.0 cm (reference position) relative to the parasternal 3rd ICS. Figure 5 (left panel) shows, that starting from quite variable resting values (tending to be higher in forearm position + 27.5 cm and − 27.5 cm), vascular resistance failed to decrease significantly with exercise at 15% MVC in the forearm position + 27.5 cm, thus reaching significantly higher resistance values as compared to the positions 0.0 cm (reference position) or − 27.5 cm. The latter both, in contrast, showed highly significant resistance decreases with exercise. To cover a certain anatomical/ physiological range of 10 mmHg in local venous outflow pressure at the level of 0.0 cm (reference position) relative to the 3rd parasternal ICS, resistance values for 0.0 cm (reference position) were calculated alternatively for an assumed venous outflow pressure of 10 mmHg (instead of 0 mmHg). This alternative estimation did not affect the finding of a significant higher vascular resistance when working at 15%MVC in forearm position + 27.5 compared to 0.0 cm (reference position, + 10 mmHg venous outflow pressure). With static exercise at 20% MVC (Fig. 5, right panel), these postural differences disappeared in line with FBF readings (Fig. 5, upper middle panel) which was lower after 1, but not after 2 min, of exercise in the forearm position of + 27.5 cm compared to 0.0 cm (reference position) or − 27.5 cm.

In an explorative approach, no significant bivariate correlations were found between MICT at 15% or 20% MVC and intra- or post-exercise peak FBF in any test position. In term, neither MICT nor peak FBF was related to potentially relevant factors of local endurance (see Introduction section) such as forearm maximal muscle CSA (in total or as involved in handgrip), forearm H_2_0 displacement volume, age, BMI, CFC (as a non-invasive parameter of capillary exchange surface), resting FBF, post-occlusion peak FBF, or venous compliance with a likely coincidental exception of a correlation (r = − 0.563, p = 0.045) between MICT at 20% MVC with 0.0 cm reference position and venous compliance.

## Discussion

We presently tested the hypothesis that local isometric handgrip endurance may be impaired when elevating the forearm to 27.5 cm above atrial level where gravitational blood flow limitations may occur physiologically. Though experimental arterial occlusion at the upper arm reportedly decreases MICT at < 40% MVC, Rohmert’s curve has not been studied with free circulation exposed to low local intravascular hydrostatic pressure^3,4,6,12–15,26,27^.

### Effect of forearm elevation

As a main finding, forearm elevation to + 27.5 cm above the 3rd ICS (0.0 cm, reference position) significantly reduced MICT at 15% MVC by a functionally relevant time span of > 1 min, while no such significant effect was observed with 20%, 30% 40% or 70% MVC. In line with our hypothesis, forearm elevation to + 27 cm above reference level significantly and massively decreased exercise FBF throughout the initial 4 min at 15% MVC and the 1st min at 20% MVC and this was also true for individual peak FBF. This significant FBF limitation also persisted when comparing excess blood flow (increases from resting values), although resting values were already significantly lower at forearm position + 27.5 cm compared to 0.0 cm. Notably, with further increases in MVC to 30%, 40%, and 70% FBF levels and peak FBF revealed no such positional differences and were found progressively reduced, however, they did not reach zero in line with^6^. The latter might be explained by contributions from skin and fat blood flow as well as from muscle areas with incomplete vascular compression (see study limitations).

Though these findings may readily be expected from studies showing massively impaired MICT with incomplete or complete arterial occlusion^4,12,14,25–27^, the presently reported gravitational/hydrostatic effects occur physiologically at free circulation, with limb elevation and involve considerably different hemodynamic conditions. Experimental partial arterial/venous occlusion similarly reduces arterial inflow pressure but at the same time increases venous outflow pressure, such that the latter equals the pressure of occlusion cuff which acts as an outlet resistance^40^. This increase in venous outflow pressure may counteract an upstream venular collapse and circulatory arrest caused through contraction-induced intramuscular pressure increase and, in addition, shift venous collapse/resistance from small to more proximal large cuff-compressed veins, which both may lead to muscle blood flow increase^32,41^. In contrast, under the presently studied conditions of limb elevation, venous outflow pressure drops to (not lower than or remains) around zero resulting in local venous collapse not observed with proximal venous occlusion. This should result in a postcapillary resistance increases, which despite arteriolar vasodilation^28,42^ decreases limb blood flow, thereby limiting capillary pressure drop and tissue dehydration^28,31,43,44^. Thus, limb elevation should facilitate contraction-induced vessel compression and blood flow reduction with consequences for MICT.

In line with this concept, we indeed presently found a significantly higher vascular resistance in position + 27.5 cm vs. 0.0 cm after 2 min of handgrip at 15% MVC (starting from an already slightly higher resting level). This finding was significant irrespective of whether we assumed venous outflow pressure at the control level of 3rd ICS to be zero or 10 mmHg (with the latter scenario allowing for venous return at positive central venous pressure). Thus, at low levels of %MVC with high FBF responses, elevation of the statically exercising limb to position + 27.5 cm from 0.0 cm, hemodynamic adjustment to metabolic demand and thus MICT may be limited by two factors: (a) Increase in local, most likely post-capillary, vascular resistance and (b) larger hydrostatic pressure drop in arterial than venous conduit vessels. In line with this concept, such postural differences in intra-exercise FBF as well as peak FBF disappeared together with those in MICT beyond 20% (except for 40% MVC). Also, lower levels of pre-exercise resting and post-exercise peak/recovery FBF in the elevated forearm position with 15% and 20% MVC may be attributable to these two factors (a) and (b)^31,32^. Whether such postural hemodynamic limitations can affect muscle recovery from fatiguing exercise warrants further studies that, in contrast to the present study, should involve identical work position before resuming different recovery positions. More generally, one might speculate that gravitational/hydrostatic hemodynamic limitation may potentially play a role in shorter MICT of shoulder compared to leg and lower trunk muscles^5,45^, in the frequent occupational fatigue and pain of neck and shoulder muscles, or in MICT limitations of highly trained rock climbers^46^. Hemodynamic limitations of MICT appear understudied also in aging and clinical conditions with endothelial dysfunction, low muscle mass and related risk of falls^9,19,22,47–52^. However, one has to keep in mind, that a higher %MVC is needed for a given motor task when muscle mass and absolute MVC are reduced.

### Effects of forearm lowering

Moreover, our study also yields new information on whether MICT and related hemodynamics are affected by a limb work position well below the atrial level. Obviously, Rohmert’s curve at position − 27.5 cm compared to 0.0 cm remains unchanged and, furthermore, blood flow as well as calculated resistance do not significantly differ between these two forearm positions. Unlike the situation of limb elevation (see above), lowering the limb to below atrial level increases the hydrostatic component of local arterial inflow and venous outflow pressure to similar extent—provided that dynamic muscle pump is absent. Thereby, autoregulative and veno-arteriolar reflexes responses may prompt local arteriolar vasoconstriction among other mechanisms limiting increases in blood flow, capillary pressure and related transcapillary fluid losses^28,29,40,42,53,54^. Upon isometric contraction, the integral FBF exercise response to all % MVC under test also did not significantly differ between position − 27.5 and 0.0 cm despite some putative differences in pressure and resistance distribution. Interestingly, post-exercise peak FBF was, however, significantly lower with forearm position − 27.5 cm compared to 0.0 cm for all levels of %MVC under test. The factors behind this obviously transient difference in post-exercise FBF remain speculative and beyond the scope of this discussion.

### Study limitations

Non-invasive VOP has remained a reference method for repetitive measurement of segmental FBF and been validated for isometric forearm exercise^4,35,36^. However, as a limitation, it includes all muscles beside skin and fat within the chosen limb segment. Because handgrip work at defined %MVC levels may differentially involve fingers and wrist flexors as well as, to some extent, extensors, their variable contribution in individual %MVC may have caused blood flow responses ranging from several-fold increases to complete compression-induced arrest. Thus, the integral segmental FBF response assessed by VOP does not reliably reflect such local maxima or minima. Accordingly, instead of a complete circulatory arrest (expected in theory with vessel compression at about > 40% MVC) a rather moderate decline in exercise FBF (maxima) from 15% MVC to 70% was observed in line with Humphreys and Lind^6^, which does not exclude regional complete ischemia or regional blood flow increases. However, as to be derived from the higher post-exercise than intra-exercise peak FBF, a %MVC-related vessel compression during static work clearly limited FBF adjustment to metabolic demand of the muscle portion involved in handgrip work in differential hydrostatic conditions as discussed above.

Repetitive fatiguing forearm isometric exercise tests, as presently performed on 4 consecutive study days, may be associated with early transient loss in MVC or some long-term gain in muscle mass and MVC, not only at high but potentially also at low to moderate intensity of 20–50%MVC involving blood flow restrictions as used by athletes and in clinical rehabilitation to facilitate muscle hypertrophy^55^. MVC changes occurring within a single test day (from pre-to post-test), or between any of the 4 test days (Table 4) were below 6% and controlled for by adjustment of %MVC to each test-day’s actual pre-test MVC values. As important, careful randomization of the five %MVC levels and three forearm position (see Methods section) should have minimized the impact of longitudinal MVC changes, whereby the left-to-right test order alternated between subjects.

The present study did not include EEG or EMG measures of central or muscle fatigue. Prolonged low-intensity isometric contractions of around 15%MVC may involve central as well as peripheral fatigue^56,57^ increasing perceived effort with only minor effects on MVC^20,58^. Moreover, isometric contraction at 20% MVC may enhance corticospinal excitability (likely via group III/IV afferents) when combined with ischemia by brachial arterial occlusion, which leads to MICT decreases^26^. The role of central fatigue in the shorter MICT with elevated working position therefore presently remains open, given that the significant blood flow restrictions in this working position may affect motoneuron output^26^. Central fatigue could, however, compromise reaction time, attention and task vigilance as required for maintenance of relative handgrip force within the ± 2.5% range presented by a feedback tracker. To ensure correct MICT assessment without EMG, intolerance to continue isometric handgrip at any %MVC level despite vocal encouragement by the supervisor was ensured by a BORG scale rating > 19. Notably, the rather large significant differences of > 1 min in MICT between the position + 27.5 cm and the positions 0.0 cm or − 27.5 cm can hardly be explained by variability in tolerance to local pain/exhaustion or task vigilance and most likely reflects peripheral physiological limitations. However, the lack of an objective measure of complete fatigue apart from task failure may qualify our study as preliminary and hypothesis-generating.

As another shortcoming, the present data are limited to men and related studies in women are definitely needed. Gender-specific investigations are warranted regarding the impact of age, muscle mass, training intervention and other physiological factors on MICT. Since we presently studied a young healthy male group with normal vascular risk factors, IMT and forearm (micro-) vascular function under fasting conditions, the effect of vascular or metabolic risk remains open. 

## Conclusions

Elevating forearm position to well above atrial level significantly decreases FBF during light static exercise via reduction in both, perfusion pressure as well as conductance. This is associated with a considerably impaired local static endurance in terms of MICT at 15 %MVC where exercise blood flow is found increased. Whether such postural MICT reduction are causally linked to such gravitational/hydrostatic FBF limitations remains to be clarified. Notably, lowering the forearm position to below atrial level with increased local hydrostatic pressures, in contrast, does not affect exercise FBF or MICT at any % MVC.

## Abbreviations

BMI: Body mass index
CSA: Cross-sectional area
CFR: Capillary filtration rate
CFC: Capillary filtration capacity
ECG: Electrocardiogram
FBF: Forearm blood flow
IMT: Intima-media-thickness
MICT: Maximal isometric contraction time
MVC: Maximal voluntary contraction
VOP: Venous occlusion plethysmography

## Declarations

### Data availability

The data sets used and/or analyzed during the present study are available from the corresponding author on reasonable request.
